# Effect of polyunsaturated fatty acids supplementation on nutritional status in patients with pancreatic cancer: a systematic review and meta-analysis

**DOI:** 10.7717/peerj.21323

**Published:** 2026-05-20

**Authors:** Guizhi Liu, Chongcong Wu, Xiaoyue Yang, Zicheng Liu, Yonglong Zhu, Ting Yang, Jiangping Zhang, Xiaoling Lin

**Affiliations:** 1 Center for Reproductive Medicine, The Fifth Affiliated Hospital of Sun Yat-sen University, Zhuhai, China; 2 Department of Nursing, The Fifth Affiliated Hospital of Sun Yat-sen University, Zhuhai, China; 3 School of Nursing, Xiamen Medical College, Xiamen City, China

**Keywords:** Pancreatic cancer, n-3 polyunsaturated fatty acids, Eicosapentaenoic acid, Docosahexaenoic acid, Nutritional status, Cancer cachexia, Systematic review, Meta-analysis

## Abstract

**Background:**

Patients with pancreatic cancer frequently experience malnutrition that is often unresponsive to conventional nutritional interventions.

**Objective:**

To evaluate the impact of n-3 polyunsaturated fatty acids (PUFAs), specifically eicosapentaenoic acid (EPA) and docosahexaenoic acid (DHA), supplementation on body weight (BW), lean body mass (LBM), body mass index (BMI), serum albumin (ALB), and serum prealbumin (PA) in this population. This systematic review was prospectively registered with PROSPERO (CRD42023402888).

**Methods:**

Reviewers searched PubMed, Embase, Cochrane Library, Web of Science, Scopus, and ProQuest from inception to 31 January 2026. Risk of bias was assessed using the Cochrane Risk of Bias Tool (RoB 1.0). Statistical analyses were performed using RevMan 5.4. Certainty of evidence was evaluated using GRADEpro GDT.

**Results:**

Nine randomized controlled trials (RCTs) were included and analyzed. Risk of bias was low in two studies, unclear in six, and high in one. The meta-analysis indicated that n-3 PUFAs may produce modest weight gain (SMD 1.41, 95% CI [0.36–2.46]; *P* = 0.008; low certainty, *I*^2^ = 94%), though clinical significance is uncertain. No statistically significant effects were observed on LBM (SMD 0.21, 95% CI [−0.38–0.81]; *P* = 0.48; moderate certainty), BMI (WMD 0.59, 95% CI [−0.42–1.60]; *P* = 0.25; moderate certainty), ALB (SMD −0.07, 95% CI [−0.81–0.66]; *P* = 0.84; low certainty), or PA (SMD 0.13, 95% CI [−0.18–0.43]; *P* = 0.42; moderate certainty).

**Conclusion:**

This meta-analysis suggests that n-3 PUFAs may produce modest weight gain, but evidence is insufficient to support meaningful improvement in overall nutritional status. Due to low certainty, substantial heterogeneity, and small sample sizes, recommendations remain individualized rather than routine. Well-designed, large-scale trials are needed to clarify which patients may benefit from this intervention.

## Introduction

Pancreatic cancer represents a major global public health challenge, characterized by high mortality, poor prognosis, and rapidly increasing incidence (Siegel, Miller & Jemal, 2017; Ilic & Ilic, 2016). Worldwide incidence has doubled over the past two decades, with projections indicating that it will soon become the second leading cause of cancer death in Western countries (Klein, 2021; Stoffel, Brand & Goggins, 2023). Globally, pancreatic cancer is the seventh most common cause of cancer-related death among both men and women (Sung et al., 2021), and it is anticipated to surpass breast cancer as the third leading cause of cancer death across Europe (Ferlay, Partensky & Bray, 2016).

As pancreatic cancer progresses, 80–90% of patients develop malnutrition characterized by wasting, fatigue, and weight loss that frequently advances to severe cachexia (Johal et al., 2022). This condition is typically refractory to conventional nutritional interventions (Gärtner et al., 2016; Miller et al., 2022). Malnutrition and cachexia compromise tolerance to surgery and adjuvant therapies, exacerbating treatment-related complications while diminishing quality of life and survival. Chemotherapy-induced toxicities—notably nausea and vomiting—further exacerbate these deficits, perpetuating a vicious cycle (Gilliland et al., 2017; Basile et al., 2019). Malnutrition and cachexia in pancreatic cancer patients are associated with prolonged hospitalization, reduced treatment responsiveness, and elevated mortality (Martin et al., 2017; Barber et al., 1999; Braga et al., 1998; Braga et al., 2004). Given that nearly all cancer patients require some form of nutritional intervention (Segura et al., 2005), and considering its established role as frontline therapy (Fan et al., 2023), timely nutritional support represents a fundamental requirement for all pancreatic cancer patients, particularly those presenting with malnutrition or cachexia (Pezzilli et al., 2020).

Polyunsaturated fatty acids (PUFAs) are straight-chain fatty acids containing two or more double bonds, with carbon chain lengths ranging from 18 to 22 carbon atoms. Based on double bond location, they are classified into n-3, n-6, n-7, and n-9 series, of which n-3 and n-6 are most prevalent. Although the registered title refers broadly to PUFAs, this review specifically focuses on n-3 PUFAs, given their distinct anti-inflammatory properties and prevalent use in cancer nutrition research. The n-3 series comprises essential fatty acids—including alpha-linolenic acid (ALA; 18:3n-3), eicosapentaenoic acid (EPA; 20:5n-3), and docosahexaenoic acid (DHA; 22:6n-3)—that exhibit anti-inflammatory and immunomodulatory properties (Freitas & Campos, 2019; Watanabe & Tatsuno, 2017). The n-6 series, exemplified by arachidonic acid (AA; 20:4n-6), generates pro-inflammatory eicosanoids (Zirpoli et al., 2020) and was not a primary intervention in the included studies.

n-3 PUFAs exhibit immunomodulatory, anti-inflammatory, and anticancer properties, and they are increasingly used as nutritional supplements in cancer therapy (Freitas & Campos, 2019; Watanabe & Tatsuno, 2017). Randomized trials have shown that multimodal interventions enriched with n-3 PUFAs stabilize body weight in pancreatic cancer patients (Solheim et al., 2017). Barber et al. (2001) further demonstrated that n-3 PUFA-enriched supplements reverse weight loss in cachectic patients, attenuating cachexia progression. Similarly, Werner et al. (2017) reported that an 8-week multimodal intervention providing two cans daily of n-3-enriched formula increased dietary intake, body weight, and lean mass in this population, concomitantly improving quality of life. However, these findings remain inconsistent. Suzuki et al. (2010) found that EPA supplementation did not significantly improve weight in 60 cachectic cancer patients compared with placebo. Similarly, Solheim et al. (2017) reported that n-3 PUFA supplementation had no significant effect on quality of life in pancreatic cancer patients and was associated with adverse events including appetite loss and diarrhea. Moses et al. (2004) further observed that n-3 PUFA-containing supplements did not significantly alter body weight or lean mass in this population. Moreover, few studies have explicitly differentiated between the effects of n-3 and n-6 PUFAs or stratified their analyses accordingly—a critical limitation given that the relative contribution of these subtypes may significantly influence nutritional and inflammatory outcomes.

Currently, findings from the limited number of randomized controlled trials (RCTs) examining whether n-3 PUFA supplementation improves nutritional status in pancreatic cancer patients remain inconsistent. Only two relevant systematic reviews are available: one did not evaluate the effects of n-3 PUFA supplementation on nutritional status (Miller et al., 2022), and the other focused on various nutritional interventions for cachexia, malnutrition, and weight loss but lacked comprehensive assessment of nutritional outcome measures (Emanuel et al., 2022).

Therefore, this systematic review and meta-analysis aims to assess the impact of n-3 PUFA supplementation on nutritional status indicators (body weight (BW), lean body mass (LBM), body mass index (BMI), serum albumin (ALB), and serum prealbumin (PA)) in pancreatic cancer patients to provide more reliable evidence for clinical practice.

## Materials and methods

This systematic review and meta-analysis is reported according to the PRISMA 2020 guidelines and the Grading of Recommendations Assessment, Development and Evaluation (GRADE) approach of the Cochrane Collaboration (Page et al., 2021; Cumpston et al., 2019). The study was registered on PROSPERO (registration number: cRD42023402888).

### Search strategy

Two reviewers (GZL and CCW) independently searched six electronic databases including PubMed, Embase, Cochrane Library, Web of Science, Scopus, and ProQuest from their inception to 31 January 2026. Relevant references were traced, and grey literature was manually searched on Open Grey. Search terms included “Pancreatic Neoplasms”, “Fatty Acids, Unsaturated”, “n-3 polyunsaturated fatty acids”, “EPA”, and “DHA”. The detailed search strategy is presented in Table 1, per the PubMed database.

**Table 1:** Search strategy on PubMed.

#1	(((((((((((((((((Pancreatic Neoplasms[MeSH Major Topic]) OR (Neoplasm, Pancreatic)) OR (Pancreatic Neoplasm)) OR (Pancreas Neoplasms)) OR (Neoplasm, Pancreas)) OR (Neoplasms, Pancreas)) OR (Pancreas Neoplasm)) OR (Neoplasms, Pancreatic)) OR (Cancer of Pancreas)) OR (Pancreas Cancers)) OR (Pancreas Cancer)) OR (Cancer, Pancreas)) OR (Cancers, Pancreas)) OR (Pancreatic Cancer)) OR (Cancer, Pancreatic)) OR (Cancers, Pancreatic)) OR (Pancreatic Cancers)) OR (Cancer of the Pancreas)
#2	(((((((((((((((((((((((((Fatty Acids, Omega-3[MeSH Major Topic]) OR (Omega-3 Fatty Acid)) OR (Acid, Omega-3 Fatty)) OR (Fatty Acid, Omega-3)) OR (Omega 3 Fatty Acid)) OR (Omega-3 Fatty Acids)) OR (n-3 Oil)) OR (Oil, n-3)) OR (n 3 Oil)) OR (n3 Oil)) OR (Oil, n3)) OR (n-3 Fatty Acids)) OR (n 3 Fatty Acids)) OR (Omega 3 Fatty Acids)) OR (n3 PUFA)) OR (PUFA, n3)) OR (n3 Polyunsaturated Fatty Acid)) OR (n3 Oils)) OR (n-3 Oils)) OR (n 3 Oils)) OR (N-3 Fatty Acid)) OR (Acid, N-3 Fatty)) OR (Fatty Acid, N-3)) OR (N 3 Fatty Acid)) OR (n-3 Polyunsaturated Fatty Acid)) OR (n 3 Polyunsaturated Fatty Acid)
#3	(((((((((Eicosapentaenoic Acid[MeSH Major Topic]) OR (EPA)) OR (5,8,11,14,17-Eicosapentaenoic Acid)) OR (Eicosapentanoic Acid)) OR (Acid, Eicosapentanoic)) OR (omega-3-Eicosapentaenoic Acid)) OR (omega 3 Eicosapentaenoic Acid)) OR (Timnodonic Acid)) OR (Icosapent)) OR (5,8,11,14,17-Icosapentaenoic Acid)
#4	((((((((((((((((((Docosahexaenoic Acids[MeSH Major Topic]) OR (DHA)) OR (Acids, Docosahexaenoic)) OR (Docosahexenoic Acids)) OR (Acids, Docosahexenoic)) OR (Docosahexaenoic Acid)) OR (Acid, Docosahexaenoic)) OR (Docosahexaenoic Acid (All-Z Isomer))) OR (Docosahexaenoic Acid Dimer (All-Z Isomer))) OR (Docosahexaenoic Acid, 3,6,9,12,15,18-Isomer)) OR (Docosahexaenoic Acid, 4,7,10,13,16,19-(All-Z-Isomer))) OR (Docosahexaenoic Acid, Sodium Salt)) OR (Docosahexaenoic Acid, 4,7,10,13,16,19-(All-Z-Isomer), Cesium Salt)) OR (Docosahexaenoic Acid, 4,7,10,13,16,19-(All-Z-Isomer), Potassium Salt)) OR (Docosahexaenoic Acid, 4,7,10,13,16,19-(Z,Z,Z,Z,Z,E-Isomer))) OR (Docosahexaenoic Acid, 4,7,10,13,16,19-Isomer)) OR (Docosahexaenoic Acid, 4,7,10,13,16,19-Isomer, Sodium Salt)) OR (Docosahexaenoate)) OR (Docosahexaenoic Acid, 4,7,10,13,16,19-(All-Z-Isomer), Cerium Salt)
#5	(((((((((((Fatty Acids, Unsaturated[MeSH Major Topic]) OR (Acids, Unsaturated Fatty)) OR (Unsaturated Fatty Acids)) OR (Unsaturated Fatty Acid)) OR (Acid, Unsaturated Fatty)) OR (Fatty Acid, Unsaturated)) OR (Polyunsaturated Fatty Acids)) OR (Acids, Polyunsaturated Fatty)) OR (Fatty Acids, Polyunsaturated)) OR (Polyunsaturated Fatty Acid)) OR (Acid, Polyunsaturated Fatty)) OR (Fatty Acid, Polyunsaturated)
#6	(((#2) OR (#3)) OR (#4)) OR (#5)
#7	(#1) AND (#6)

### Inclusion and exclusion criteria

Inclusion and exclusion criteria were based on the PICOS framework.

#### Inclusion criteria

(1) Study population (P): Patients aged ≥18 years diagnosed with pancreatic cancer, including those undergoing surgery, radiotherapy, chemotherapy, or presenting with cachexia;

(2) Intervention (I): Nutritional supplementation enriched with n-3 PUFAs;

(3) Comparator (C): Conventional, isonitrogenous, and isocaloric nutritional support;

(4) Outcome measures (O): Primary outcomes were changes from baseline in ALB, PA, BW, and LBM. Secondary outcomes included adverse events (*e.g.*, thrombocytopenia, appetite loss, infections);

(5) Study design (S): RCTs.

#### Exclusion criteria

(1) Non-randomized study designs (*e.g.*, reviews, meta-analyses, systematic reviews, conference abstracts, protocols, case reports, animal studies);

(2) Duplicate publications or data;

(3) Studies with unavailable full texts or unextractable data;

(4) Studies using healthy subjects as comparators.

### Study selection

Two reviewers (GZL and CCW) independently screened the titles and abstracts, assessed full-text articles of potentially eligible studies, and reached consensus on eligibility. Disagreements were resolved through discussion or by consultation with a third reviewer (XLL). The screening process involved duplicate removal, title/abstract screening, full-text assessment, and final eligibility determination.

### Data extraction

Data were extracted independently by two reviewers (GZL and CCW) using a pilot-tested standardized form. Extracted information included study identifiers (first author, country, year), participant characteristics (age, sex, sample size, disease stage), intervention specifics (dosage, frequency, duration), comparator description, and outcome results. For continuous variables (BW, LBM, ALB, PA), we extracted means, standard deviations (SDs), and sample sizes. Where SDs were unavailable, we estimated them from standard errors, confidence intervals, or interquartile ranges using established formulas. For adverse events, we extracted the number of events and total participants in each group. Disagreements were resolved by consensus or referral to a third reviewer (XLL).

### Risk of bias and quality assessment

Two reviewers (GZL and CCW) independently assessed risk of bias using the Cochrane Risk of Bias Tool (RoB 1.0) (Higgins et al., 2011). Disagreements were resolved through discussion or by consultation with a third reviewer (XLL). The following domains were evaluated: sequence generation, allocation concealment, blinding of participants and personnel, blinding of outcome assessment, incomplete outcome data, selective reporting, and other bias. Each item was judged as “low risk”, ”high risk”, or ”unclear”.

### Data analysis

All meta-analyses were conducted using RevMan 5.4 software (Cochrane Collaboration). For dichotomous outcomes, we calculated relative risk (RR) or odds ratio (OR) with 95% confidence intervals (CIs); for continuous outcomes, we used standardized mean difference (SMD) or weighted mean difference (WMD), also with 95% CIs. We assessed statistical heterogeneity using Cochran’s Q test and quantified it with *I*^2^ statistics. We considered heterogeneity substantial when *P* < 0.10 for the Q test or *I*^2^ ≥ 50%, in which case we applied a random-effects model; otherwise, we used a fixed-effects model. To explore sources of heterogeneity, we performed subgroup and sensitivity analyses where appropriate. For outcome domains including at least five studies, we examined publication bias by visual inspection of funnel plots.

### GRADE evidence quality assessment

Two reviewers (GZL and CCW) independently assessed the certainty of evidence using GRADEpro GDT, with discrepancies resolved through discussion or by a third reviewer (XLL) if consensus was not reached. In accordance with GRADE methodology, evidence from RCTs began as high-certainty evidence and was subsequently downgraded for concerns in any of five domains: risk of bias, inconsistency, indirectness, imprecision, and publication bias.

## Results

### Study identification and selection

**Figure 1:**
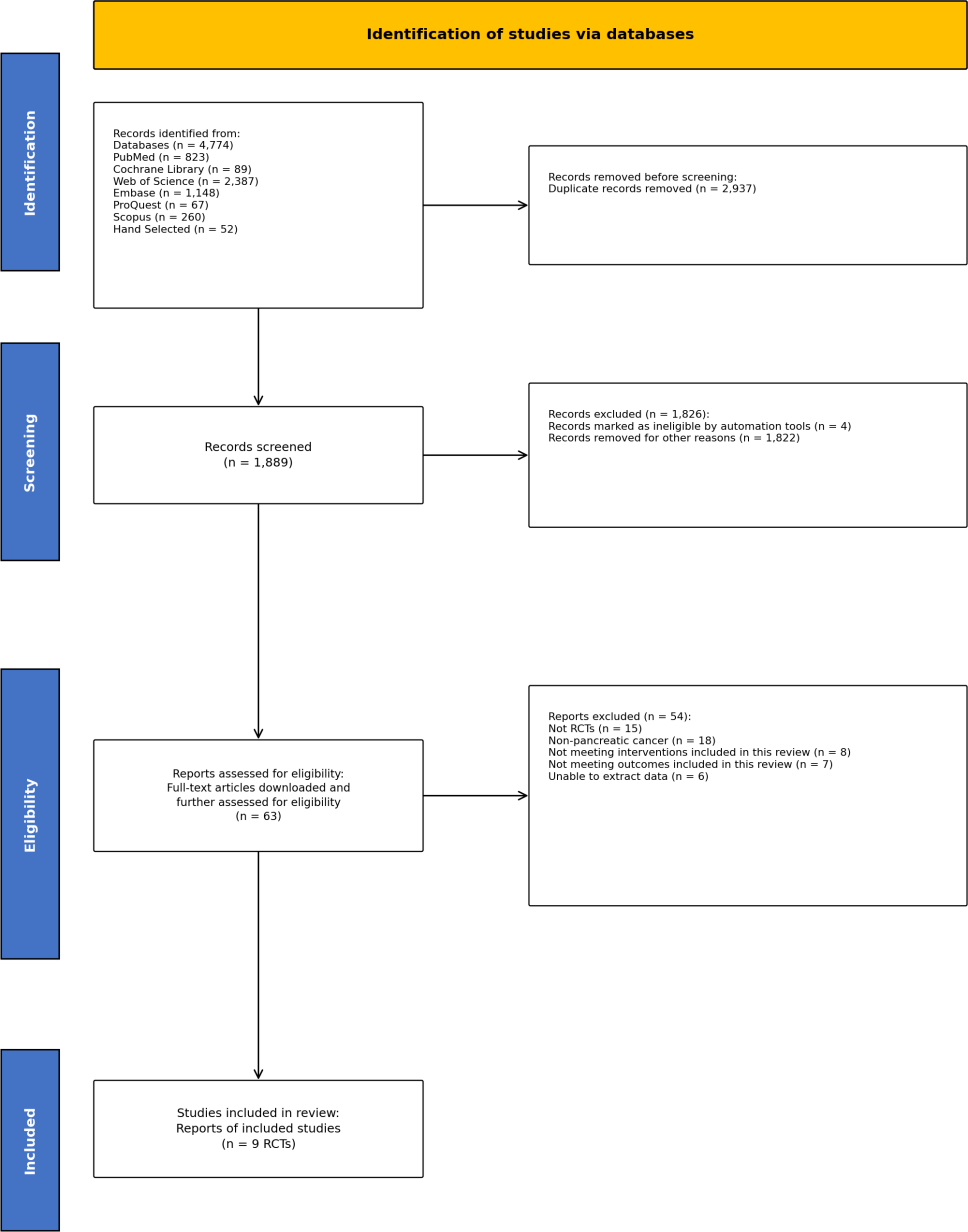
PRISMA flowchart of RCTs assessing the effectiveness of n-3 polyunsaturated fatty acid supplementation on nutritional status in patients with pancreatic cancer. RCT, randomized controlled trial.

Database searches yielded 4,826 records. After deduplication using NoteExpress (*n* = 2,937), 1,889 records underwent title and abstract screening, of which 1,826 were excluded for the following reasons: irrelevant topic, review or conference abstract, case report, or animal study. Sixty-three records were assessed for eligibility in full text, of which nine RCTs met inclusion criteria and were included in the meta-analysis. The study selection process is illustrated in Fig. 1.

### Risk of bias in included studies

**Figure 2:**
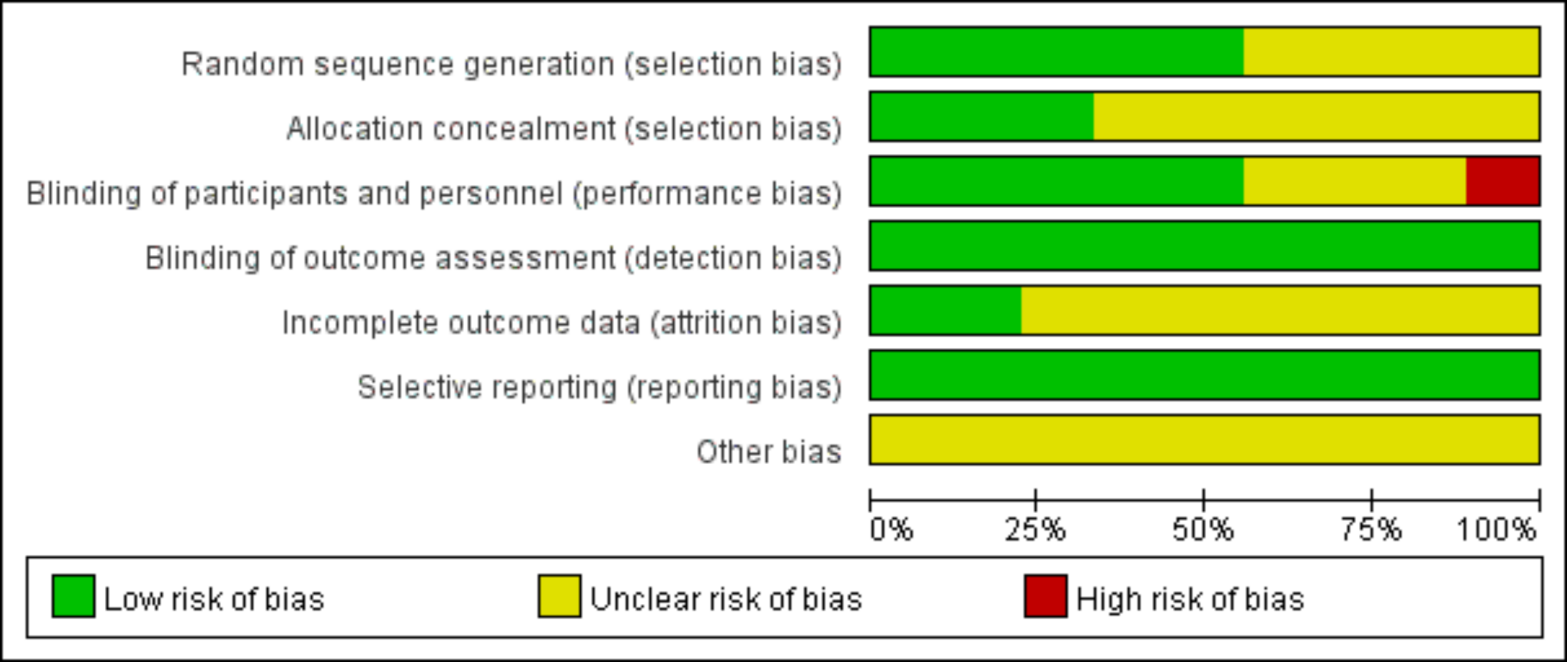
Risk of bias proportion diagram for included studies.

**Figure 3:**
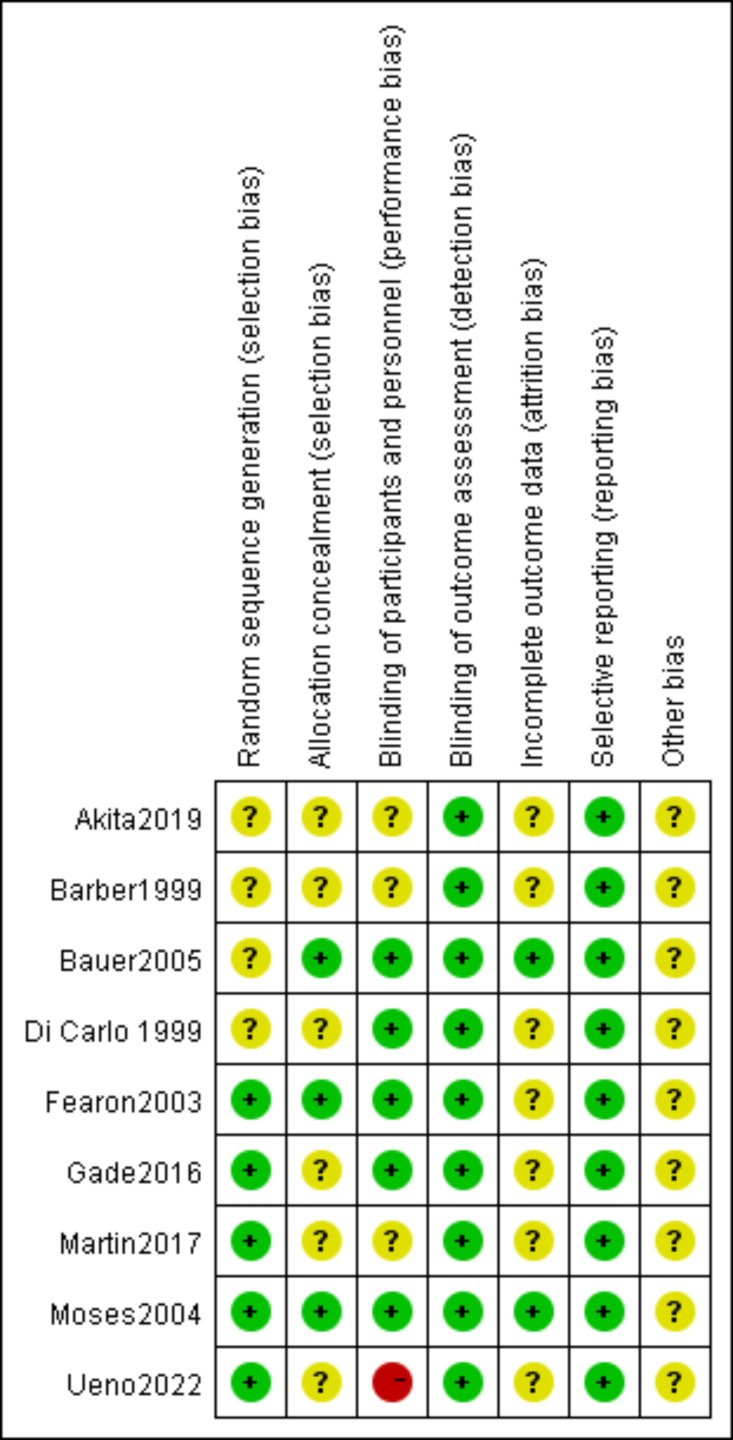
Summary of risk of bias assessment for included studies.

Risk of bias assessments are summarized in Figs. 2 and 3. Two studies showed low risk, six showed unclear risk, and one showed high risk.

Random sequence generation. Three studies reported computer-generated randomization (Fearon et al., 2003; Moses et al., 2004; Ueno et al., 2022). One study described a single-center, parallel-group design with balanced 1:1 randomization (Gade et al., 2016). One study randomized patients based on their ability to implement supplementation (Martin et al., 2017). The remaining four studies did not specify the randomization method (Di Carlo et al., 1999; Barber et al., 1999; Bauer et al., 2005; Akita et al., 2019).

Allocation concealment. Three studies used sequentially numbered, sealed envelopes containing computer-generated assignments, with randomization performed by an independent third party (Fearon et al., 2003; Moses et al., 2004; Bauer et al., 2005). These three studies were reports of the same center randomized trial; Moses et al. (2004) was a secondary analysis, and Bauer et al. (2005) was a *post-hoc* analysis, both maintaining the original randomization and blinding procedures. The remaining six studies did not describe allocation concealment methods.

Blinding of participants and personnel. Three studies used a double-blind design (Fearon et al., 2003; Moses et al., 2004; Bauer et al., 2005). Two studies were open-label (Gade et al., 2016; Ueno et al., 2022). The remaining four studies did not specify blinding methods. Although Gade et al. (2016) was open-label and may have introduced performance bias, we judged the risk to be low because the primary outcome (BW) was objectively measured and unlikely to be influenced by the subjective intentions of participants or investigators. Conversely, we assessed Ueno et al. (2022) as high risk due to the inclusion of subjective outcomes (*e.g.*, appetite loss), which are susceptible to influence by participants or investigators.

Blinding of outcome assessment. Two studies reported blinded outcome assessment (Fearon et al., 2003; Moses et al., 2004). The remaining seven studies were open-label or partially blinded. All included studies reported objective outcome measures (BW, LBM, BMI, ALB, PA, thrombocytopenia, and neutropenia). Although blinding of outcome assessment was not universally implemented, the risk of detection bias was judged to be low owing to the objective nature of these outcomes.

Incomplete outcome data. Two studies were judged to be at low risk of attrition bias. Moses et al. (2004) used a rigorous double-blind RCT design with predefined sample size calculations and balanced attrition patterns across groups. Bauer et al. (2005) used advanced statistical methods (generalized estimating equations) to handle missing data in a *post-hoc* analysis of a large RCT. The remaining seven studies did not report methods for handling missing data; however, this was unlikely to have influenced the results given the objective nature of the reported outcome measures (BW, LBM, BMI, ALB, PA, thrombocytopenia, and neutropenia).

Selective reporting and other biases. Two studies were registered in clinical trial registries (UMIN000033589; Akita et al., 2019 and UMIN000003658) (Ueno et al., 2022). The remaining seven studies did not report trial registration. All studies reported prespecified outcomes, and no evidence of selective outcome reporting was identified. No other potential sources of bias were identified.

### Characteristics of the included studies

Nine RCTs from six countries (United Kingdom, Japan, United States, Australia, Italy, and Denmark) were included. All were RCTs with clearly defined eligibility criteria and parallel-group designs. Publication years: 1999–2022. Participants: adults with pancreatic cancer; sample sizes 24–200 (median 66). All nine studies used n-3 PUFAs (EPA/DHA). EPA dosages ranged 1.1–2.18 g/day; three studies did not report dosage. Duration varied: 5 days to 8 weeks. See Table 2 for detailed study characteristics.

**Table 2:** Characteristics of included studies.

**Study (First author, year)**	**Country**	**Age (mean ± SD)**	**Sample size/ male/female**	**Intervention (dosage, frequency, duration)**	**Control**	**Outcomes**
Barber et al. (1999)	United Kingdom	T: 64 ± 2.5; C: 60 ± 4.0	T: 18/NA/NA; C:18/NA/NA	EPA 2.18 g + DHA 0.92 g/day, 1 time/d, 3 weeks	Conventional nutritional support	①②③
Akita et al. (2019)	Japan	T: 67.8 ± 10.7; C: 66.4 ± 9.8	T: 31/11/20; C: 31/16/15	n-3 PUFAs (dose NR), 2 times/d, 5 weeks	Conventional nutritional support	①②④⑥⑦
Martin et al. (2017)	United States	T: 60 ± 13.5; C: 62 ± 7.0	T: 44/20/24; C: 27/12/15	n-3 PUFAs (dose NR), 3 times/d, 5 days	Conventional nutritional support	①②④⑧
Fearon et al. (2003)	United Kingdom	T: 67 ± 9.7; C: 67 ± 10.2	T: 95/54/41; C: 105/56/49	EPA 1.5 g/day, 2 times/d, 8 weeks	Isocaloric supplement	③⑤
Moses et al. (2004)	United Kingdom	T: 65 ± 6.0; C: 70 ± 11.6	T: 9/6/3; C: 15/4/11	EPA 1.5 g/day, 2 times/d, 8 weeks	Isocaloric supplement	③⑤
Bauer et al. (2005)	Australia	T: 66.8 ± 9.3; C: 68.3 ± 10.9	T: 87/NA/NA; C: 98/NA/NA	EPA 1.5 g/day, 2 times/d, 8 weeks	Isocaloric supplement	③⑤
Gade et al. (2016)	Denmark	T: 68 ± 7.8; C: 69 ± 6.5	T: 19/7/12; C: 16/10/6	n-3 PUFAs (dose NR), 1–4 times/day, 1 week	Conventional nutritional support	③
Ueno et al. (2022)	Japan	T: 68 ± 8.8; C: 69 ± 10.5	T: 43/22/21; C: 23/17/6	EPA 1.056 or 2.112 g/day, 1–2 times/day, duration NR	Conventional nutritional support	⑥⑦⑧
Di Carlo et al. (1999)	Italy	T: 63 ± 13.1; C: 61 ± 12.0	T: 33/20/13; C: 35/23/12	n-3 PUFAs (dose NR), frequency NR, duration NR	Conventional nutritional support	⑧

**Notes.**

T, treatment group; C, control group; NR, not reported; n-3 PUFAs, n-3 polyunsaturated fatty acids; EPA, eicosapentaenoic acid; DHA, docosahexaenoic acid.

Outcomes: ① serum albumin; ② serum prealbumin; ③ body weight; ④ body mass index; ⑤ lean body mass; ⑥ thrombocytopenia; ⑦ decreased appetite; ⑧ infection.

### Meta-analysis

#### Body weight (BW)

**Figure 4:**
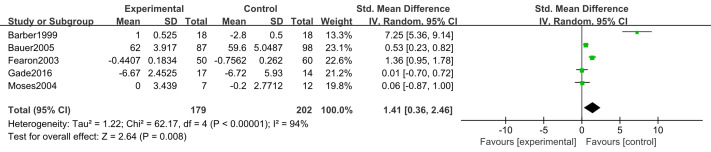
Forest plot of the effect of n-3 PUFA supplementation on BW in patients with pancreatic cancer.

Five studies (Barber et al., 1999; Moses et al., 2004; Bauer et al., 2005; Gade et al., 2016; Fearon et al., 2003) reported changes in body weight after supplementation with n-3 PUFAs. These trials included 381 participants (179 in the intervention group and 202 in the control group). Because weight change was measured using different scales, we used standardized mean difference as the effect measure. Substantial heterogeneity was present (*I*^2^ = 94%); therefore, a random-effects model was applied. Meta-analysis showed that n-3 PUFA supplementation may produce modest weight gain in pancreatic cancer patients (SMD 1.41, 95% CI [0.36–2.46]; *P* = 0.008, low certainty) (Fig. 4).

#### Lean body mass

**Figure 5:**
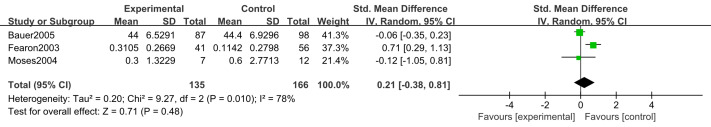
Forest plot of the effect of n-3 PUFA supplementation on LBM in patients with pancreatic cancer.

Three studies (Fearon et al., 2003; Moses et al., 2004; Bauer et al., 2005) involving 301 participants examined effects of n-3 PUFA supplementation on LBM in pancreatic cancer patients. The intervention comprised 135 participants, and the control group comprised 166 participants. Because LBM was measured using different methods, we used SMD as the effect measure. Substantial heterogeneity was present (*P* = 0.01, *I*^2^ = 78%); therefore, a random-effects model was applied. Meta-analysis showed no statistically significant effect (SMD 0.21, 95% CI [−0.38–0.81]; *P* = 0.48, moderate certainty) (Fig. 5).

Sensitivity analysis by systematic exclusion of individual studies indicated that removing Fearon et al. (2003) substantially reduced heterogeneity (*P* = 0.90, *I*^2^ = 0%); a fixed-effect model of the remaining studies yielded SMD −0.06 (95% CI [−0.34–0.21]; *P* = 0.65). The null finding was insensitive to exclusion of individual studies.

#### BMI changes

**Figure 6:**
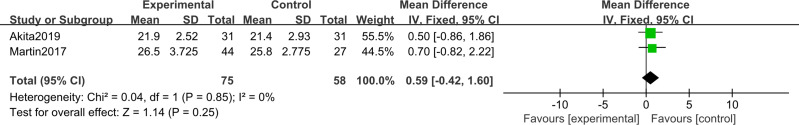
Forest plot of the effect of n-3 PUFA supplementation on BMI in pancreatic cancer patients.

Two studies (Martin et al., 2017; Akita et al., 2019) including 133 participants (75 in the intervention group and 58 in the control group) reported BMI changes. Because measurement units were consistent across studies, we used WMD as the effect measure. No substantial heterogeneity was observed (*P* = 0.85, *I*^2^ = 0%); therefore, a fixed-effects model was applied. Meta-analysis showed no meaningful effect on BMI (WMD 0.59, 95% CI [−0.42–1.60]; *P* = 0.25, moderate certainty) (Fig. 6).

#### Serum albumin and prealbumin

Three studies (Barber et al., 1999; Martin et al., 2017; Akita et al., 2019) involving 169 participants examined effects of n-3 PUFA supplementation on ALB and PA levels. The intervention comprised 93 participants, and the control group comprised 76 participants. Because ALB and PA were measured using different methods, we used SMD as the effect measure.

**Figure 7:**
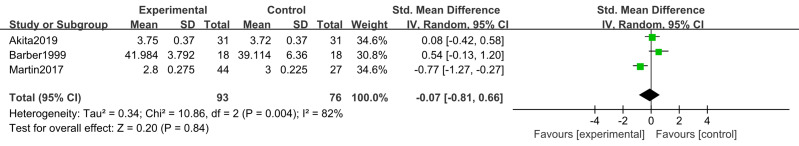
Forest plot of the effect of n-3 PUFA supplementation on ALB levels in pancreatic cancer patients.

ALB. Substantial heterogeneity was present (*I*^2^ = 82%); therefore, a random-effects model was initially applied. Meta-analysis showed no statistically significant effect (SMD −0.07, 95% CI [−0.81–0.66]; *P* = 0.84, low certainty) (Fig. 7). Sensitivity analysis excluding Martin et al. (2017) reduced heterogeneity (*P* = 0.28, *I*^2^ = 13%) and yielded SMD 0.24 (95% CI [−0.16–0.64]; *P* = 0.23); the null finding remained unchanged.

**Figure 8:**
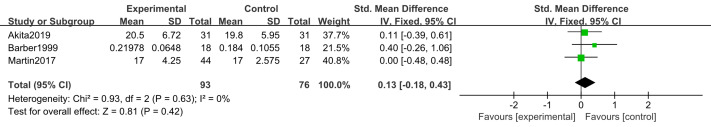
Forest plot of the effect of n-3 PUFA supplementation on PA levels in pancreatic cancer.

PA. No heterogeneity was observed (*I*^2^ = 0%); therefore, a fixed-effects model was applied. Meta-analysis showed no statistically significant effect (SMD 0.13, 95% CI [−0.18–0.43]; *P* = 0.42, moderate certainty) (Fig. 8).

#### Adverse events (thrombocytopenia, appetite loss, infection)

**Figure 9:**
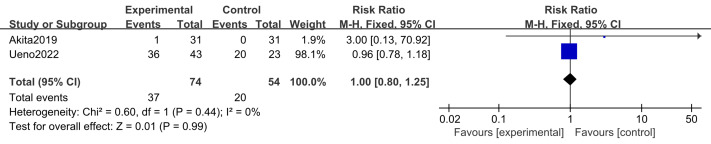
Forest plot of the effect of n-3 PUFA supplementation on thrombocytopenia in pancreatic cancer patients.

**Figure 10:**
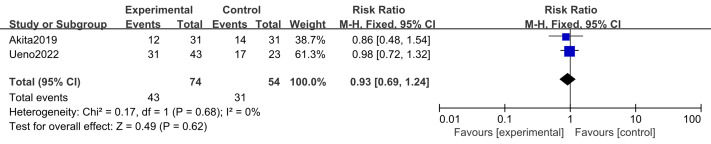
Forest plot of the effect of n-3 PUFA supplementation on appetite loss in pancreatic cancer patients.

**Figure 11:**
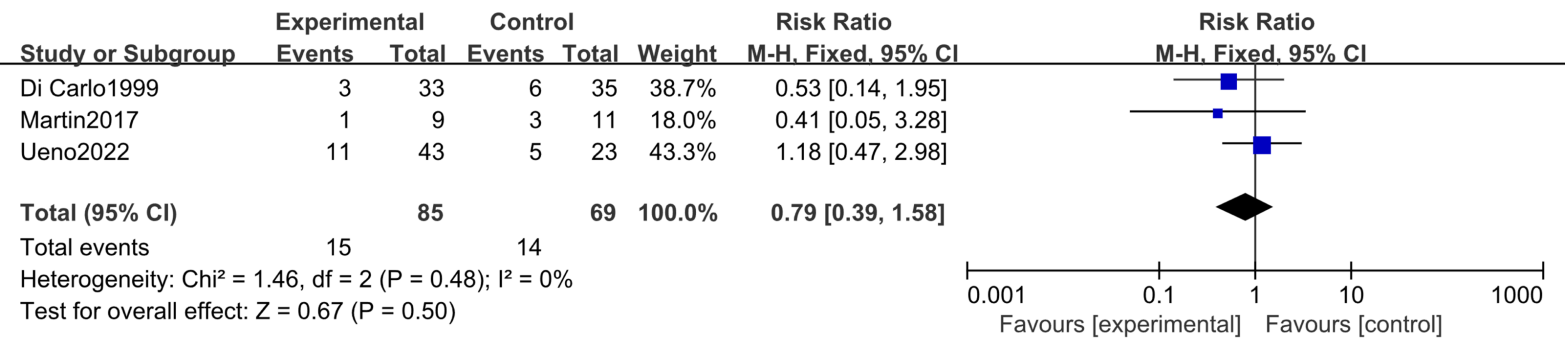
Forest plot of the effect of n-3 PUFA supplementation on infection in pancreatic cancer patients.

Two studies (Akita et al., 2019; Ueno et al., 2022) including 128 participants (74 in the intervention group and 54 in the control group) reported thrombocytopenia and appetite loss. Three studies (Di Carlo et al., 1999; Akita et al., 2019; Ueno et al., 2022) including 154 participants (85 in the intervention group and 69 in the control group) examined infection rates. No substantial heterogeneity was observed (*I*^2^ = 0%); therefore, a fixed-effects model was applied. Meta-analysis showed that n-3 PUFA supplementation had no significant effect on the incidence of thrombocytopenia (RR 1.00, 95% CI [0.80–1.25]; *P* = 0.99), appetite loss (RR 0.93, 95% CI [0.69–1.24]; *P* = 0.62), or infection (RR 0.79, 95% CI [0.39–1.58]; *P* = 0.50) (Figs. 9–11).

#### Subgroup analysis

To further elucidate the impact of n-3 PUFAs on nutritional parameters (BW, ALB, PA) in pancreatic cancer patients, we conducted subgroup analyses stratified by intervention duration (≤3 weeks *vs.* >3 weeks), intervention components (EPA alone *vs.* EPA+DHA), and intervention frequency (2 times/d *vs.* >2 times/d or <2 times/d).

##### Intervention duration.

**Figure 12:**
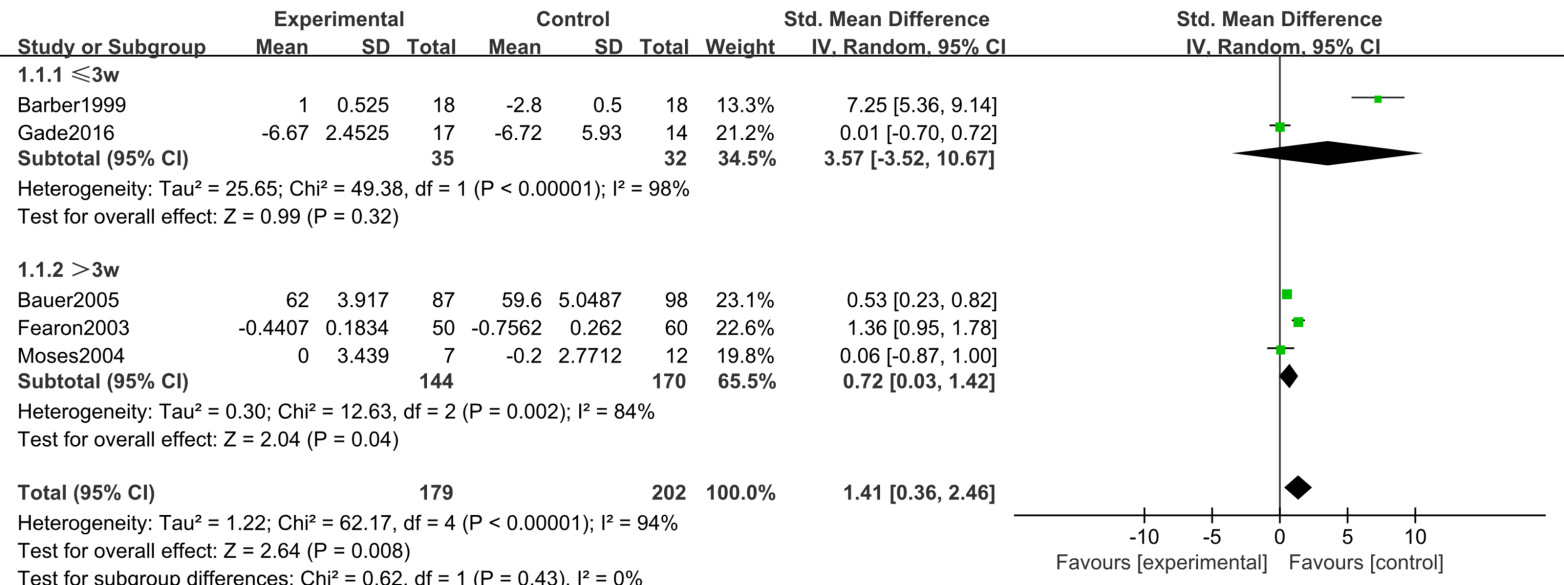
Forest plot of the effect of different intervention durations on BW in pancreatic cancer patients.

BW. Substantial heterogeneity was observed between subgroups (*P < 0.001*, *I*^2^= 94%). Using a random-effects model, the >3 weeks subgroup demonstrated a statistically significant effect (SMD 0.72, 95% CI [0.03–1.42]; *P* = 0.04), consistent with the overall pooled estimate (*P* = 0.008). Conversely, the ≤3 weeks subgroup showed no statistically significant effect. Heterogeneity was lower in the >3 weeks subgroup (*I*^2^ = 84%) compared to the ≤3 weeks subgroup (Fig. 12).

**Figure 13:**
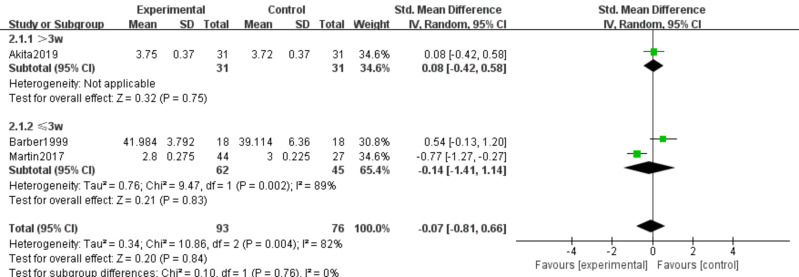
Forest plot of the effect of different intervention durations on ALB levels in pancreatic cancer patients.

ALB. No significant subgroup differences were detected (*P* = 0.84; *I*^2^ = 82%). The pooled effect was not statistically significant for both >3 weeks and ≤3 weeks subgroups (SMD −0.07, 95% CI [−0.81–0.66]) (Fig. 13).

**Figure 14:**
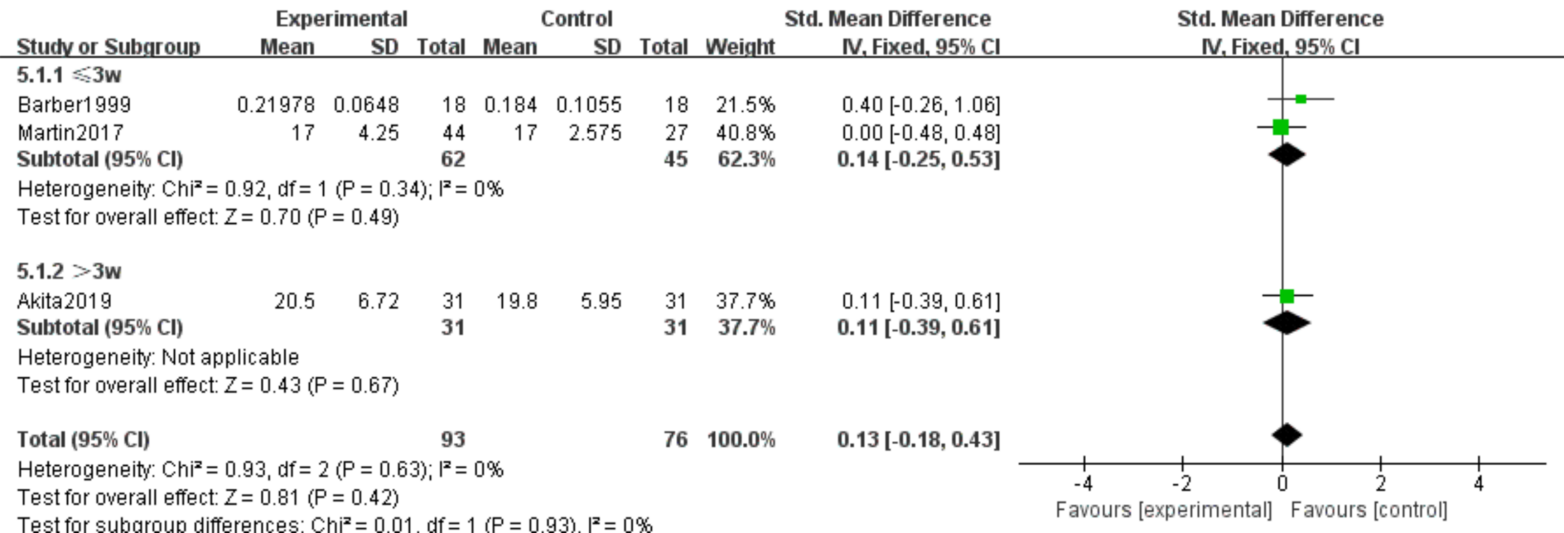
Forest plot of the effect of different intervention durations on PA levels in pancreatic cancer patients.

PA. No subgroup heterogeneity was observed (*P* = 0.42; *I*^2^ = 0%). Fixed-effects analysis revealed no statistically significant difference between duration subgroups (SMD 0.13, 95% CI [−0.18–0.43]) (Fig. 14).

##### Intervention components.

**Figure 15:**
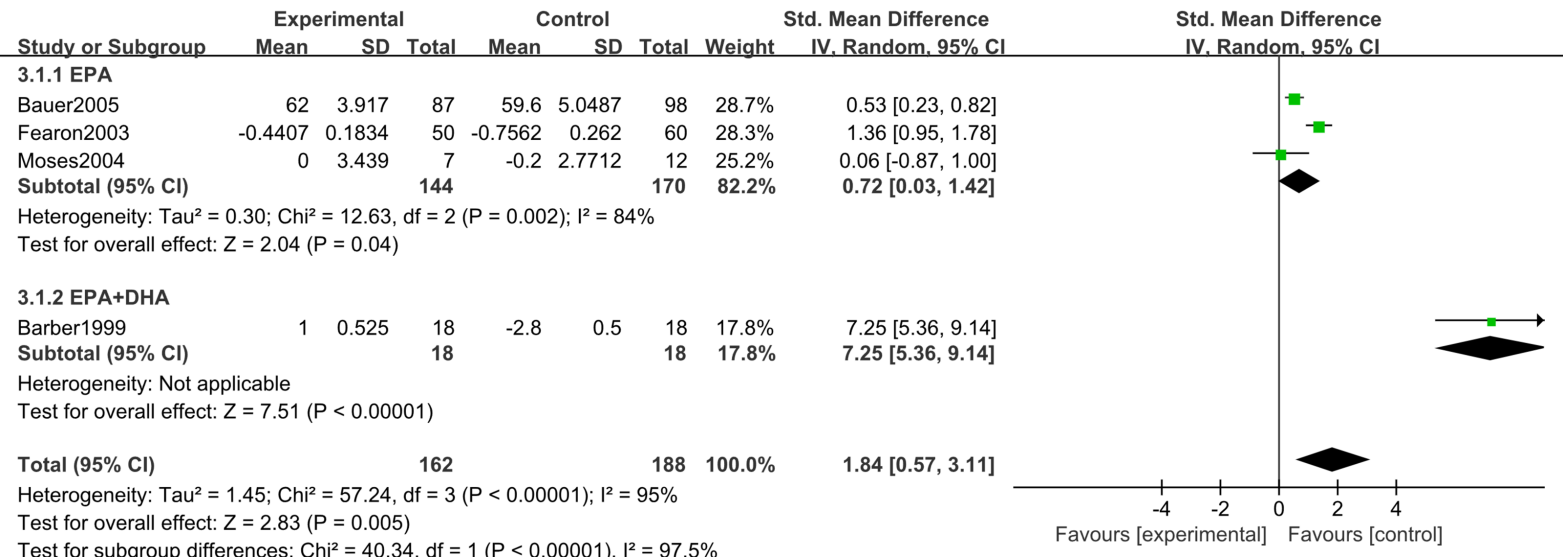
Forest plot of the effect of different intervention components on the nutritional status of pancreatic cancer patients.

For BW, substantial heterogeneity existed between EPA-only and EPA+DHA subgroups (*P* = 0.005; *I*^2^ = 95%). The EPA-only subgroup retained substantial heterogeneity (*I*^2^ = 84%), though 11% lower than the overall estimate. Random-effects analysis yielded a pooled SMD 0.72 (95% CI [0.03–1.42]; *P* = 0.04), with effect estimates favoring the intervention in both subgroups (Fig. 15).

##### Intervention frequency.

**Figure 16:**
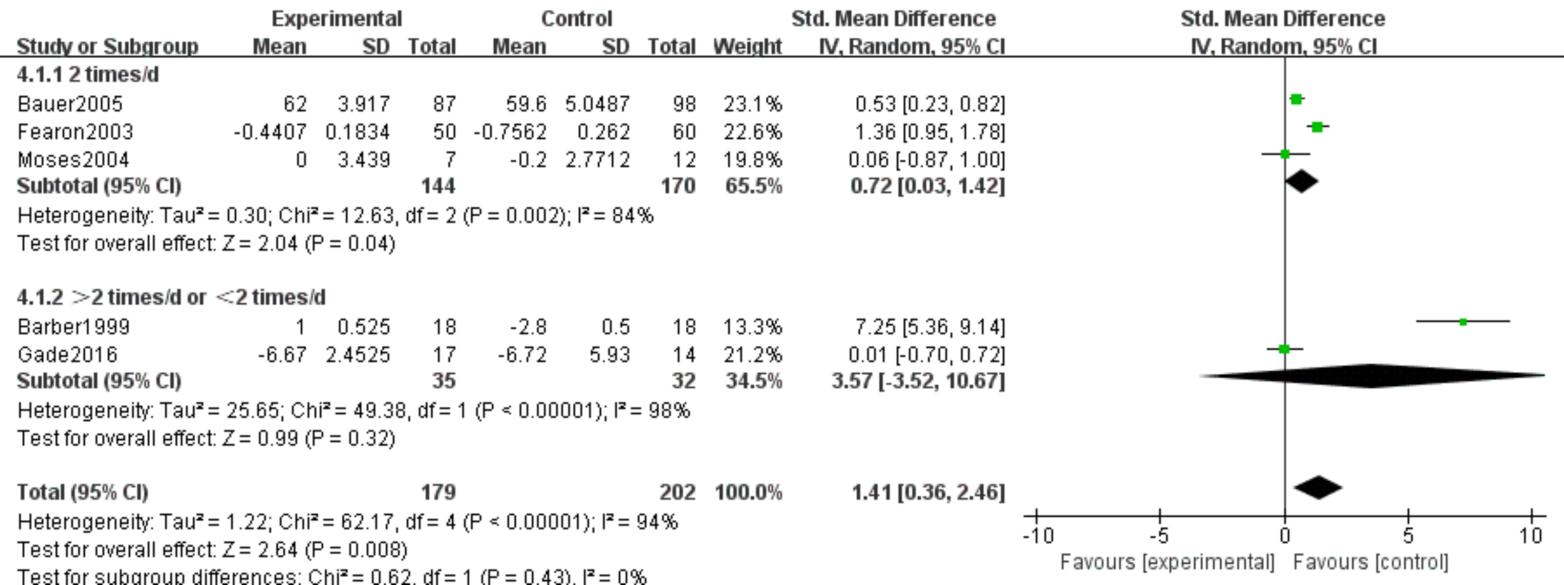
Forest plot of the effect of different intervention frequencies on the BW of pancreatic cancer patients.

BW. Substantial heterogeneity was observed between subgroups (*P* < 0.001; *I*^2^ = 94%). Using a random-effects model, the 2 times/d subgroup demonstrated a statistically significant effect (SMD 0.72, 95% CI [0.03–1.42]; *P* = 0.04), consistent with the overall pooled estimate (SMD 1.41, 95% CI [0.36–2.46]; *P* = 0.008). Heterogeneity was lower in the 2 times/d subgroup (*I*^2^ = 84%) compared to the combined >2 times/d or <2 times/d subgroup (*I*^2^ = 98%), which showed no statistically significant effect (SMD 3.57, 95% CI [−3.52–10.67]; *P* = 0.32) (Fig. 16).

**Figure 17:**
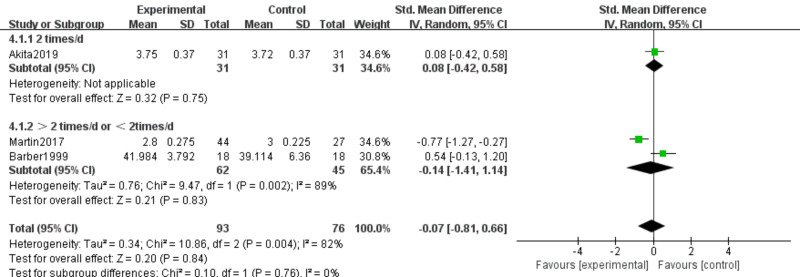
Forest plot of the effect of different intervention frequencies on the ALB of pancreatic cancer patients.

ALB. No significant subgroup differences were detected (*P* = 0.84; *I*^2^ = 82%). Using a random-effects model, the pooled effect was not statistically significant across all frequency categories (SMD −0.07, 95% CI [−0.81–0.66]) (Fig. 17).

**Figure 18:**
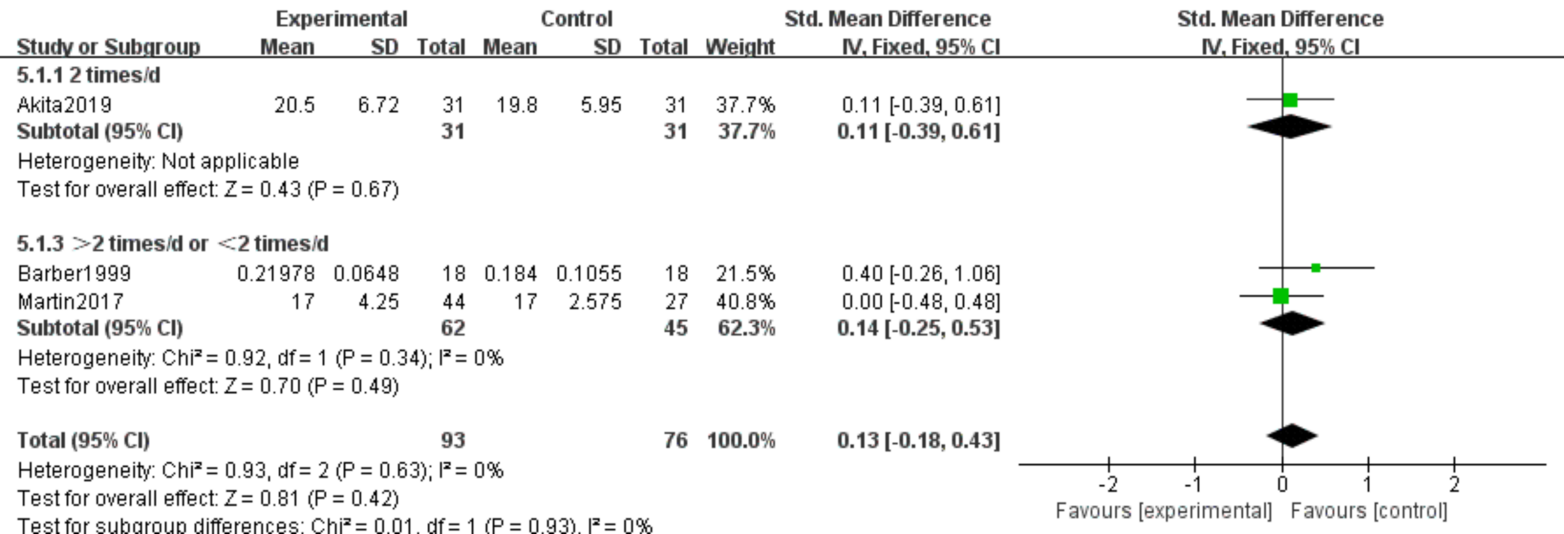
Forest plot of the effect of different intervention frequencies on the PA of pancreatic cancer patients.

PA. Absence of subgroup heterogeneity (*P* = 0.42; *I*^2^ = 0%) supported use of a fixed-effects model, showing no statistically significant differences among subgroups (SMD 0.13, 95% CI [−0.18–0.43]) (Fig. 18).

### Analysis of publication bias

**Figure 19:**
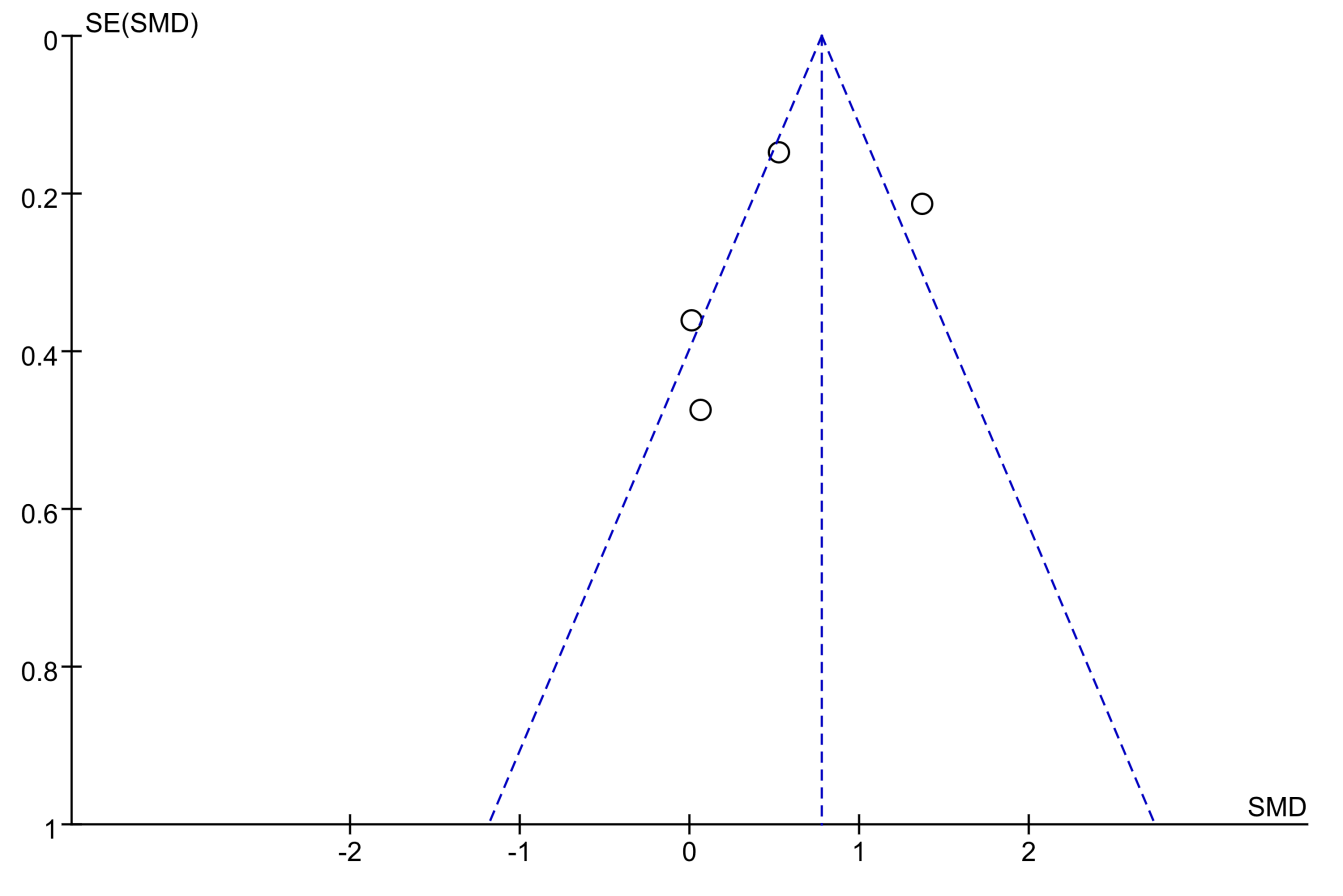
Funnel plot of the effect of n-3 PUFA on body weight in pancreatic cancer patients.

Given that fewer than 10 studies were available for each primary outcome, there was limited statistical power to detect publication bias using funnel plots. We therefore generated funnel plots only for outcomes with at least 5 studies. Visual inspection revealed a broadly symmetrical distribution around the pooled effect estimate, with most studies falling within the expected funnel region and only one study lying outside this area (Fig. 19). This pattern suggests minimal evidence of publication bias, though the small number of studies precludes definitive conclusions.

### GRADE assessment of evidence certainty

Evidence certainty was evaluated using the GRADE framework (Table 3). For BW, substantial heterogeneity (*I*^2^ = 94%) resulted in low certainty evidence. LBM and BMI were rated as moderate certainty due to inconsistency and imprecision, respectively. ALB was downgraded for both inconsistency (*I*^2^ = 82%) and imprecision, yielding low certainty. PA was downgraded for imprecision only, resulting in moderate certainty. No downgrades were applied for risk of bias or indirectness.

**Table 3:** Summary of GRADE evidence quality for different outcome measures.

**Outcome measures**	**Number of studies**	**Study design**	**Risk of bias**	**Inconsistency**	**Indirectness**	**Imprecision**	**Sample size** ** (** **T/C** **)**	**Combined effect size**	**95% confidence interval**	**Evidence quality**
BW	5	RCT	Not serious	Very serious	Not serious	Not serious	179/202	1.41	(0.36, 2.46)	⊕⊕∘∘
LBM	3	RCT	Not serious	Serious	Not serious	Not serious	135/166	0.21	(−0.38, 0.81)	⊕⊕⊕∘
BMI	2	RCT	Not serious	Not serious	Not serious	Serious	75/58	0.59	(−0.42, 1.60)	⊕⊕⊕∘
ALB	3	RCT	Not serious	Serious	Not serious	Serious	93/76	−0.07	(−0.81, 0.66)	⊕⊕∘∘
PA	3	RCT	Not serious	Not serious	Not serious	Serious	93/76	0.13	(−0.18, 0.43)	⊕⊕⊕∘

**Notes.**

T, treatment group; C, control group; BW, body weight; LBM, lean body mass; BMI, body mass index; ALB, serum albumin; PA, serum prealbumin; RCT, randomized controlled trial. Evidence quality ratings: ⊕⊕⊕⊕, high; ⊕⊕⊕∘, moderate; ⊕⊕∘∘, low; ⊕∘∘∘, very low.

## Discussion

Studies indicate that n-3 PUFA supplementation benefits cancer patients’ nutritional status, modulates immune function, and reduces postoperative complications (Ma et al., 2015; Miller et al., 2022; Emanuel et al., 2022). This review examined the effects of n-3 PUFAs on nutritional indicators (BW, LBM, BMI, ALB, and PA) and adverse events in pancreatic cancer patients.

### Body weight

Our findings suggest that n-3 PUFA supplementation may produce modest BW gain in pancreatic cancer patients, though the evidence is of low certainty due to substantial heterogeneity (*I*^2^ = 94%) and small sample sizes. This possibility is suggested by prior systematic reviews (Bauer et al., 2005; Ma et al., 2015), but the clinical significance remains uncertain. Limited evidence suggests possible improvements in nutritional status and weight stabilization (Daly et al., 1995; Werner et al., 2017; Solheim et al., 2017) and partial attenuation of weight loss (Barber et al., 2000; Barber et al., 2001; Davidson et al., 2004; Abe et al., 2018). Proposed mechanisms include provision of energy substrates and modulation of catabolic mediators involved in cachexia (Barber et al., 2001), though these pathways remain inadequately characterized in pooled analyses.

Previous trials report weight gains of 0.5–2.3 kg following EPA supplementation. However, these studies were small, methodologically heterogeneous, and often lacked statistical significance, precluding definitive conclusions (Barber et al., 1999; Wigmore et al., 2000; Barber et al., 2000; Moses et al., 2004) interpretation. Therefore, any interpretation should remain cautious and individualized.

### Lean body mass and BMI

Abe et al. (2018) reported modest increased skeletal muscle mass in pancreatic cancer patients following 4 and 8 weeks of n-3 PUFA supplementation, though these findings were derived from small, short-term studies (Kocher et al., 2020). Delpino & Figueiredo (2022) observed a 0.17 kg increase in LBM with n-3 supplementation, though between-group differences were not statistically significant and BMI remained unchanged. Barber et al. (2000) reported a 0.75 kg increase in LBM after 3 weeks with 2 g/day EPA. Bauer & Capra (2004) noted a clinically relevant but non-statistically significant improvement in LBM (median 4.4 kg).

In contrast, our meta-analysis found no statistically significant effect of n-3 PUFA supplementation on LBM or BMI, consistent with low certainty evidence. This discrepancy may reflect metabolic abnormalities in pancreatic cancer patients that impede lean mass accretion (Ma et al., 2015), insufficient EPA doses, or inadequate intervention duration. BMI increases require sustained intervention, whereas included trials ranged from 5 days to 8 weeks.

Short-term trials (5 days–1 week) were retained because they capture early nutritional endpoints relevant to preoperative care. However, these brief interventions assess acute-phase protein responses rather than sustained body composition changes. In neoadjuvant settings, nutritional support is often confined to 5–10 days whilst patients await surgery or chemoradiation. Observing that n-3 PUFA supplementation may influence ALB and PA trajectories—even transiently—informs this short-course practice pattern. Leave-one-out analysis showed that removing either short trial altered the pooled albumin SMD by <0.02 and reduced heterogeneity only marginally (*I*^2^ 82%→79%), confirming they add information without undue leverage. We did not apply GRADE downgrading for indirectness because our PICO question explicitly included any RCT reporting nutritional biomarkers, regardless of duration. Consequently, these ultrashort studies are not interpreted as evidence of sustained weight gain, but as indicators that n-3 PUFA supplementation may rapidly influence hepatocyte synthetic rate—an observation requiring validation in larger trials.

### Serum albumin and prealbumin

ALB is the most abundant plasma protein and a commonly used marker of nutritional status (Ward et al., 2022). Together with weight loss, it serves as a simple, economical, and reliable parameter for assessing malnutrition. Previous studies reported that PA levels modestly increased following n-3 PUFA supplementation (*P* = 0.02) (Braga et al., 2004), and EPA-enriched enteral nutrition reduced ALB decline preoperatively (Ashida et al., 2019). However, our meta-analysis found no statistically significant improvement in serum ALB or PA levels. This null finding may reflect small sample sizes, short intervention durations (5 days to 5 weeks), and underlying systemic inflammation in pancreatic cancer patients that elevates acute-phase proteins and suppresses hepatic synthesis (Siddiqui et al., 2007).

Proposed mechanisms by which n-3 PUFAs might affect these biomarkers include modulation of hepatic protein synthesis, though our pooled results do not support a clinically meaningful effect. Nutritional recovery is a chronic process; future studies should extend intervention and observation periods to better determine these effects.

### Adverse events (thrombocytopenia, appetite loss, and infections)

Di Carlo et al. (1999) suggested that n-3 PUFA-rich supplements might improve nutritional status in pancreatic cancer patients receiving neoadjuvant chemoradiotherapy. However, the fishy taste of these supplements may limit compliance and exacerbate nausea and appetite loss. Our meta-analysis found that n-3 PUFA supplementation did not significantly reduce the incidence of adverse events. This null finding may reflect small sample sizes, heterogeneous intervention doses, varied background treatments, and poor palatability limiting intake. Future research should optimize supplement formulation and conduct larger center trials.

### Heterogeneity and PUFA subtypes

Previous studies have reported inconsistent results of PUFA supplementation, particularly when differentiating between n-3 and n-6 PUFAs, which have distinct biological roles in inflammation and metabolism. Most of the included studies used marine-derived n-3 fatty acids (EPA and/or DHA), either alone or in combination with other nutrients, while n-6 PUFAs were rarely isolated or compared directly. This limits our ability to draw definitive conclusions about the differential effects of PUFA subtypes. The n-3/n-6 ratio may influence nutritional and inflammatory outcomes, though our pooled data do not permit direct assessment of this hypothesis.

Given limited studies and absence of head-to-head comparisons, our subgroup analyses remain exploratory. Observed heterogeneity may partly reflect differences in PUFA composition across trials. Future RCTs are needed to explicitly compare n-3 and n-6 PUFAs or stratify outcomes by PUFA type to elucidate differential effects.

### Strengths and limitations

Strengths. We followed PRISMA 2020 guidelines; all included studies were RCTs; and we analyzed multiple nutritional outcomes, enabling comprehensive assessment.

Limitations. Despite a possible signal of benefit, our review is constrained by substantial heterogeneity from variable supplement compositions (EPA-alone *vs.* EPA/DHA combinations, rarely with full fatty-acid ratios disclosed), divergent dosing schedules and durations (5 days–8 weeks), and non-standardized outcome assessment. BW was routinely reported, whereas LBM, ALB, and PA were measured with different methods. Many trials were small with unclear-to-high risk of bias in randomization and allocation concealment.

Implications for research. Future trials are needed to detail fatty acid profiles, adopt uniform dosing regimens aligned with international guidelines, and conduct large center RCTs using validated nutritional endpoints. Only then would such evidence clarify the role of n-3 PUFAs in attenuating nutritional status in pancreatic cancer; current certainty remains insufficient to support routine clinical recommendation.

## Conclusion

Current evidence provides limited support for a possible role of n-3 PUFA supplementation in producing modest weight gain in pancreatic cancer patients, though certainty is low and clinical significance remains uncertain. Effects on LBM and PA were not clinically meaningful, consistent with low-to-moderate certainty evidence. Although some trials reported changes in BMI, ALB, and PA, these findings were inconsistent and imprecise. Therefore, any claims of broad nutritional benefits should be interpreted with caution, and recommendations remain individualized rather than routine.

Further well-designed, large-scale trials are necessary to clarify potential benefits and risks. Until such evidence is available, nutritional interventions should remain individualized. Given current limitations, including substantial heterogeneity and small sample sizes, we cannot establish specific recommendations regarding duration, frequency, or dosage.

## Supplemental Information

10.7717/peerj.21323/supp-1 Supplemental Information 1 PRISMA checklist

## Additional Information and Declarations

### Data Availability

The following information was supplied regarding data availability: This is a systematic review/meta-analysis.
